# Hippo–YAP Pathway Dysregulation and Prognostic Implications in HPV-Negative Oropharyngeal Carcinomas

**DOI:** 10.3390/cancers18142337

**Published:** 2026-07-20

**Authors:** Ernesto Martín-Guillermo, Israel Rivera-García, Ana Álvarez-Alonso, Carlota Guizán-Alonso, Alejandra García-Torre, Daniela Corte-Torres, Corina Lorz, Juana M. García-Pedrero, Juan P. Rodrigo

**Affiliations:** 1 Department of Otolaryngology, Hospital Universitario Central de Asturias, Avda. del Hospital Universitario s/n, 33011 Oviedo, Spain; 2 Head and Neck Cancer Research Group, Instituto de Investigación Sanitaria del Principado de Asturias (ISPA), Avda. del Hospital Universitario s/n, 33011 Oviedo, Spain; israel.rivera@ispasturias.es (I.R.-G.); uo317161@uniovi.es (A.Á.-A.); uo291028@uniovi.es (C.G.-A.); 3 Instituto Universitario de Oncología del Principado de Asturias (IUOPA), University of Oviedo, c/Julian Clavería s/n, 33006 Oviedo, Spain; 4 Spanish Biomedical Research Network in Cancer (CIBERONC), Instituto de Salud Carlos III, Av. Monforte de Lemos, 3–5, 28029 Madrid, Spain; clorz@ciemat.es; 5 Maxillofacial Oncology, Instituto de Investigación Sanitaria del Principado de Asturias (ISPA), Avda. del Hospital Universitario s/n, 33011 Oviedo, Spain; 6 Biobanco del Principado de Asturias (BioPA), Instituto de Investigación Sanitaria del Principado de Asturias (ISPA), Avda. del Hospital Universitario s/n, 33011 Oviedo, Spain; 7 Biomedical Innovation Unit, CIEMAT, Av. Complutense, 40, 28040 Madrid, Spain; 8 Research Institute 12 de Octubre imas12, University Hospital 12 de Octubre, Av. de Córdoba s/n, 28041 Madrid, Spain

**Keywords:** oropharyngeal squamous cell carcinoma, head and neck carcinoma, human papillomavirus, YAP1, TAZ, survival

## Abstract

**Background:** Human papillomavirus (HPV)-unrelated oropharyngeal squamous cell carcinoma (OPSCC) is a clinically aggressive disease characterized by poor outcomes and limited prognostic biomarkers. The Hippo–YAP pathway has emerged as a key regulator of tumorigenesis in epithelial cancers, although its role in OPSCC remains incompletely defined. **Methods:** We conducted a retrospective study including 215 patients with HPV-negative OPSCC treated surgically with curative intent. Tissue microarrays were constructed, and immunohistochemical analysis of YAP1 and TAZ expression was performed. Associations with clinicopathological variables, disease-free survival (DFS) and disease-specific survival (DSS) were assessed using univariable and multivariable analyses. **Results:** Nuclear and cytoplasmic YAP1 expression were detected in 53% and 85.6% of tumors, respectively. Both nuclear and cytoplasmic YAP1 expression were significantly associated with reduced DFS (*p* = 0.019 and *p* = 0.004, respectively) and DSS (*p* = 0.01 and *p* = 0.006, respectively). Multivariable analysis identified nodal involvement (HR = 1.64, *p* = 0.023) and nuclear YAP1 expression (HR = 1.65, *p* = 0.004) as independent predictors of DFS, and advanced tumor stage (HR = 1.57, *p* = 0.038), nodal involvement (HR = 2.34, *p* < 0.001), and nuclear YAP1 expression (HR = 1.78, *p* = 0.002) as independent predictors of DSS. In contrast, nuclear TAZ expression was observed at a lower frequency (14.5%) and showed no significant association with clinical parameters or survival outcomes. **Conclusions:** YAP1 expression, particularly its nuclear activated form, emerges as an independent poor prognostic factor in HPV-negative OPSCC, whereas nuclear TAZ expression was rather infrequent and showed no clinical relevance. These findings highlight the importance of Hippo pathway dysregulation in this tumor subtype and suggest the potential of YAP1 as both a prognostic biomarker and a therapeutic target.

## Simple Summary

Oropharyngeal squamous cell carcinoma (OPSCC) unrelated to human papillomavirus (HPV) is an aggressive disease with limited prognostic biomarkers. The Hippo–YAP signaling pathway is frequently dysregulated in epithelial cancers, but its role in this OPSCC subtype remains unclear. In this study, we evaluated the expression of YAP1 and TAZ proteins in a large cohort of HPV-negative OPSCC patients and analyzed their association with clinicopathological features and survival outcomes. We found that nuclear YAP1 expression is strongly associated with poorer disease-specific survival and acts as an independent prognostic factor, while TAZ expression showed no significant clinical impact. These findings highlight the relevance of YAP1 as a prognostic biomarker and support further investigation of the Hippo–YAP pathway as a potential therapeutic target in HPV-unrelated OPSCC.

## 1. Introduction

Oropharyngeal squamous cell carcinoma (OPSCC) is a subset of head and neck squamous cell carcinomas (HNSCCs), which together represent the sixth most common group of malignancies worldwide. Within this group, OPSCC is the third most frequent anatomical site, accounting for more than 350,000 new cases and over 170,000 deaths globally in 2020 [1]. Similar to other major malignancies, the incidence of OPSCC continues to rise, with an estimated annual increase of 2–3% reported between 2015 and 2019 [1].

OPSCC can be broadly classified into two etiological categories: tumors associated with human papillomavirus (HPV) infection (p16-positive) and those unrelated to HPV (p16-negative). The latter group is predominantly linked to well-established lifestyle risk factors, particularly tobacco and alcohol consumption, as is characteristic of other HNSCC subtypes [2,3].

Carcinogenesis in HPV-negative OPSCC is driven by chronic exposure to carcinogens, leading to the progressive accumulation of genetic alterations within epithelial cells. This multistep process ultimately results in malignant transformation [4,5]. Frequently observed molecular alterations include mutations in *TP53* and deletions in the *CDKN2A* gene, which encodes the p16 protein. Additional genetic changes involve mutations in genes such as *MLL2*, *CUL3*, *NSD1*, *PIK3CA*, and *NOTCH*, as well as copy number gains affecting *EGFR*, *CCND1*, and *FGFR* [4,6].

Among the signaling pathways implicated in OPSCC pathogenesis, the Hippo–YAP pathway plays a critical role. Originally described in *Drosophila melanogaster*, this pathway functions as a key regulator of cell proliferation, apoptosis, and organ size control [7]. It acts as a mechanosensory system, integrating physical cues from the cellular environment and translating them into transcriptional responses that influence cell growth and differentiation. Moreover, the Hippo pathway is essential for early embryonic development and the maintenance of stem cell pluripotency [8,9]. Dysregulation of this pathway has been documented in a wide range of human cancers, particularly those of epithelial origin, including lung, colorectal, breast, and liver cancers [7].

The Hippo–YAP pathway comprises two principal components. The core Hippo kinase cascade includes neurofibromatosis type 2 (NF2), which activates the serine/threonine kinases MST1 and MST2. In complex with the scaffold protein SAV1, these kinases phosphorylate and activate LATS1/2 and their cofactor MOB1A/1B. This cascade results in the inactivating phosphorylation of the transcriptional coactivators YAP1 and TAZ. When the Hippo pathway is inactive, unphosphorylated YAP1 and TAZ translocate to the nucleus, where they interact with TEAD transcription factors to promote the expression of genes involved in cell proliferation and survival, thereby supporting tumorigenesis. Conversely, activation of the Hippo pathway leads to YAP1/TAZ inhibition through phosphorylation, cytoplasmic retention, ubiquitination, and proteasomal degradation [7,8].

The Hippo pathway is highly interconnected with other signaling networks, including the epidermal growth factor receptor (EGFR), insulin receptor (IR), and platelet-derived growth factor receptor (PDGFR) pathways [9]. It also interacts with the MAPK pathway, which can enhance Hippo signaling and promote YAP1/TAZ inactivation, thereby suppressing cell proliferation [7,8]. Additionally, crosstalk with the PI3K pathway has been described, particularly involving *PIK3CA* (encoding the p110α catalytic subunit), which is mutated or overexpressed in up to 55% of HNSCC cases. Evidence suggests a close association between PIK3CA/p110α activation, phosphorylation of its downstream effector pS6, and YAP1 activation [9,10,11]. Overall, the Hippo pathway functions as a tumor suppressor system that restrains the oncogenic activity of YAP1 and TAZ. Notably, its dysregulation in cancer is often driven by altered expression of upstream regulatory components rather than direct mutations in *YAP1* or *TAZ* themselves [9].

YAP1 has been extensively studied for its role in tumor progression and metastasis across multiple epithelial malignancies, including lung, colorectal, ovarian, and breast cancers. Disruption of the Hippo pathway—often through alterations in signaling cascades such as PI3K–AKT–mTOR—leads to nuclear accumulation of YAP1, which is associated with enhanced migration, invasion, metastasis, and resistance to therapy, ultimately contributing to poor clinical outcomes [12,13].

Despite advances in understanding the molecular biology of OPSCC, significant research gaps remain, particularly for HPV-negative disease. While HPV-positive OPSCC has been extensively characterized and shows a favorable prognosis with de-escalation strategies under investigation, HPV-negative OPSCC represents a distinct biological entity with poor outcomes and limited prognostic biomarkers. Current TNM staging and histological grading provide insufficient risk stratification in this population. The Hippo-YAP/TAZ pathway has emerged as a critical regulator of cancer progression in multiple tumor types, but its role in HPV-negative OPSCC remains poorly characterized. Specifically, the following gaps exist: (1) limited data on YAP1/TAZ expression patterns specifically in HPV-negative OPSCC; (2) lack of prognostic biomarkers beyond conventional clinicopathological parameters; (3) insufficient understanding of pathway dysregulation mechanisms in this disease subtype; and (4) absence of validated therapeutic targets. Therefore, comprehensive characterization of YAP1 and TAZ expression and their prognostic implications in HPV-negative OPSCC is critically needed to improve risk stratification and identify potential therapeutic targets.

The primary objective of this study was to evaluate the expression patterns of YAP1 and TAZ proteins in HPV-negative OPSCC and determine their prognostic significance for disease-specific survival. Secondary objectives were to (1) assess associations between YAP1/TAZ expression and clinicopathological characteristics; (2) analyze subcellular localization patterns (nuclear vs. cytoplasmic) and their differential prognostic implications; (3) evaluate stromal YAP1 expression in cancer-associated fibroblasts; and (4) identify independent prognostic factors through multivariable survival analysis.

## 2. Materials and Methods

### 2.1. Study Design

This study was designed as a retrospective observational investigation comprising two complementary components. First, a descriptive analysis was conducted to address the primary objective. Subsequently, an analytical approach was applied to explore associations between clinicopathological variables and immunohistochemical findings in relation to the secondary objectives.

### 2.2. Study Population

Patients with histopathologically confirmed HPV-negative oropharyngeal squamous cell carcinoma (OPSCC) who underwent primary surgical treatment at the Central University Hospital of Asturias (Oviedo, Spain) between January 2000 and December 2009 were included. Only treatment-naïve patients were considered eligible. Inclusion criteria were (1) histopathologically confirmed squamous cell carcinoma of the oropharynx (including base of tongue, tonsil, soft palate, and oropharyngeal wall); (2) HPV-negative status; (3) primary surgical treatment with curative intent performed at our institution; (4) availability of formalin-fixed paraffin-embedded tumor tissue of sufficient quality and quantity for immunohistochemical analysis; (5) complete clinicopathological and follow-up data; and (6) no prior head and neck malignancy or concurrent second primary tumor. This study included all consecutive patients with HPV-negative OPSCC treated at our institution during the study period who met the inclusion criteria, resulting in a cohort of 215 patients. While no formal “a priori” sample size calculation was performed for this retrospective observational study, post-hoc power analysis indicated that with 215 patients and an observed event rate of 55%, this cohort provides >80% power to detect a hazard ratio of 1.5 or greater for biomarker associations with survival (alpha = 0.05, two-sided test). This sample size is comparable to or larger than previous prognostic biomarker studies in p16-negative OPSCC and represents one of the largest single-center cohorts reported for this specific patient population.

All patients underwent primary surgical resection with curative intent. Neck dissection was performed according to clinical and radiological nodal status: patients with clinically positive nodes (N+) underwent therapeutic neck dissection (levels I-V or modified radical neck dissection), while patients with clinically negative nodes (N0) underwent elective neck dissection (typically levels II-IV). The extent of neck dissection was determined by the primary tumor location and clinical staging. Postoperative radiotherapy (RT) was administered to patients based on pathological risk factors including positive or close surgical margins (<5mm), perineural invasion, lymphovascular invasion, extracapsular extension, or multiple (>3) positive lymph nodes. Concurrent chemotherapy was not administered to any patient. Tumor staging was determined according to the 8th edition of the American Joint Committee on Cancer (AJCC) staging system, and clinicopathological data were retrieved from electronic medical records. Information on HPV status was available for all patients. HPV status was analyzed using p16 immunohistochemistry, high-risk HPV DNA detection by in situ hybridization, and genotyping by GP5+/6+-PCR, as previously reported [14,15].

The study protocol complied with the principles of the Declaration of Helsinki and was approved by the Institutional Ethics Committee of the Central University Hospital of Asturias (CEImPA No. 2023.018).

### 2.3. Histopathology & Immunohistochemistry (IHC)

Formalin-fixed, paraffin-embedded (FFPE) tumor samples were obtained from the Biobank of the Principality of Asturias (PT20/00161 and PT23/00077), part of the Spanish National ISCIII Platform for Biobanks and Biomodels. Tissue microarrays (TMAs) were constructed using representative areas from each tumor specimen. Specifically, three distinct tumor regions were selected for each case, excluding necrotic areas, along with samples of non-neoplastic mucosa (FFPE tonsil tissue). Hematoxylin and eosin staining was performed on selected sections to confirm histological features. Tumor grading was established according to the 2017 World Health Organization (WHO) classification criteria.

Immunohistochemical analyses were carried out following previously validated protocols [10,16]. Briefly, 3 µm-thick sections from the TMAs were mounted on Flex IHC slides (DakoCytomation, Glostrup, Denmark), deparaffinized in xylene, and rehydrated through graded alcohols. Antigen retrieval was performed using EnVision Flex Target Retrieval Solution (DakoCytomation, Glostrup, Denmark) under different pH conditions, depending on the antibody (high pH for TAZ and low pH for YAP1). Immunostaining was conducted at room temperature using an automated Dako Autostainer Plus system (DakoCytomation, Glostrup, Denmark). The primary antibodies employed were a rabbit polyclonal anti-YAP1 antibody (Invitrogen, #PA1-46189, Waltham, MA, USA; dilution 1:500) and a mouse monoclonal anti-TAZ antibody (Abcam, #ab242313, clone CL0371, Cambridge, UK; dilution 1:100). Detection was achieved using the Dako EnVision Flex+ system (DakoCytomation, Glostrup, Denmark) with diaminobenzidine as chromogen, followed by hematoxylin counterstaining. Negative controls were included by omitting the primary antibody.

Immunohistochemical evaluation was independently performed by two observers blinded to clinical data. A good correlation was observed between observers (Kappa index = 0.85). Nuclear expression of YAP1 and TAZ was assessed according to the percentage of positively stained tumor cells and categorized into three groups (0–10%, 11–50%, and >50%), which were subsequently dichotomized into negative (≤10% stained cells) and positive (>10% stained cells) for statistical analysis. Cytoplasmic YAP1 expression was evaluated based on staining intensity and classified into four categories (negative, weak, moderate, and strong), later dichotomized into low (negative/weak) and high (moderate/strong) expression. Stromal YAP1 expression in cancer-associated fibroblasts was scored on a three-tier scale (negative, weak, and strong) and similarly dichotomized into low (negative/weak) and high expression groups.

Nuclear and cytoplasmic YAP1 expression was evaluated using different but complementary scoring systems based on biological considerations. Nuclear YAP1 represents a transcriptionally active protein, and its biological significance relates to the proportion of tumor cells with nuclear translocation; therefore, we assessed the percentage of tumor cells with nuclear positivity. Cytoplasmic YAP1 represents the inactive, sequestered form of the protein, where staining intensity better reflects the overall protein level; therefore, we used a semi-quantitative intensity score (0–3+).

The 10% cutoff for nuclear YAP1 positivity was selected based on (1) previous literature in head and neck squamous cell carcinoma demonstrating that even low levels of nuclear YAP1 (>10%) correlate with pathway activation and adverse outcomes; (2) receiver operating characteristic (ROC) curve analysis for disease-specific survival, which identified 10% as an optimal threshold; and (3) biological considerations, as YAP1 nuclear translocation is a key regulatory event and even modest nuclear accumulation indicates pathway activation.

### 2.4. Statistical Analysis

Statistical analyses were performed using IBM SPSS Statistics for Windows (version 27.0.1; IBM Corp., Armonk, NY, USA). Descriptive statistics were used to summarize clinicopathological characteristics and immunohistochemical variables. Associations between categorical variables were evaluated using the chi-square test and Spearman correlation, as appropriate. Disease-specific survival (DSS) was defined as the time from initial treatment to death attributable to the disease, and disease-free survival (DFS) was defined as the time from surgery to first recurrence (local, regional, or distant) or last follow-up, and both were estimated using the Kaplan–Meier method, with comparisons performed using the log-rank test. Hazard ratios (HRs) and 95% confidence intervals (CIs) were calculated using Cox proportional hazards regression models. Variables identified as statistically significant in univariable analyses (*p* < 0.05) were considered candidates for inclusion in the multivariable model. A multivariable regression model was then constructed using a backward stepwise selection procedure, in which all candidate variables were initially entered into the model and sequentially removed based on predefined criteria (typically *p* > 0.05 for removal), while assessing changes in model fit and stability at each step. Clinical relevance and prior knowledge were also taken into account to retain variables considered important confounders, regardless of their statistical significance, to avoid model misspecification. For time-to-event outcomes, a Cox proportional hazards regression model was used. Model adequacy was evaluated using measures of goodness-of-fit and discrimination (e.g., likelihood ratio tests). Final model results were reported as adjusted hazard ratios (HRs) with 95% confidence intervals (CIs). All statistical tests were two-sided, and a *p*-value < 0.05 was considered statistically significant.

## 3. Results

### 3.1. Clinicopathological Description

A total of 215 patients were included in the study cohort, consisting predominantly of men (n = 209, 97.2%), with only six women (2.8%). All the cases received surgical resection with neck dissection: 38 cases received unilateral selective neck dissection, 58 bilateral selective neck dissection, 36 unilateral radical neck dissection, 79 radical in one side plus selective neck dissection in the other side, and 4 bilateral radical neck dissection (staged); 146 (68%) patients received postoperative RT. The mean age at diagnosis was 57.8 years (median, 58 years). The median follow-up duration was 20 months (mean, 33.68 months) for the whole series and 65 months (mean, 74.6 months) for surviving patients, with a broad range spanning from 0 to 216 months. Data regarding tobacco and alcohol exposure were available for 211 patients. Among these, 208 (96.7%) were current or former smokers, while only three individuals (1.4%) reported no history of smoking. A comparable distribution was observed for alcohol consumption, with 208 patients identified as regular drinkers and three as abstinent. At the last follow-up, 118 patients (54.9%) had died due to tumor progression, whereas 42 (19.5%) had died from causes unrelated to the disease. The overall mortality rate reached 74.4%, with a disease-specific mortality of 54.9% attributed to OPSCC.

Tumor characteristics indicated a predominance of advanced disease at diagnosis. According to the TNM classification, T4a tumors were most frequent (n = 77, 35.8%), followed by T3 tumors (n = 74, 34.4%). Nodal involvement was common, with N2 stage observed in 108 patients (50.2%). Consequently, the majority of patients presented with advanced-stage disease, with stage IV accounting for 158 cases (73.5%), followed by stage III (n = 36, 16.7%), stage II (n = 17, 7.9%), and stage I (n = 4, 1.9%). Histopathological evaluation revealed that 96 tumors (44.7%) were well differentiated, 82 (38.1%) moderately differentiated, and 37 (17.2%) poorly differentiated. Surgical margins were free in 178 patients (83%) and microscopically involved in 37 (17%).

### 3.2. Immunohistochemical Analysis of YAP1 and TAZ Expression

Positive nuclear YAP1 expression (>10% of tumor cells) was detected in 114 tumor samples (53%). Cytoplasmic YAP1 expression showed strong intensity (+3) in 37 cases (17.2%), moderate intensity (+2) in 60 (27.9%), weak intensity (+1) in 87 (40.5%), and no expression in 31 cases (14.4%). After dichotomization, 118 cases (55%) were categorized as weak/negative and 97 (45%) as moderate/strong expression.

**Figure 1:**
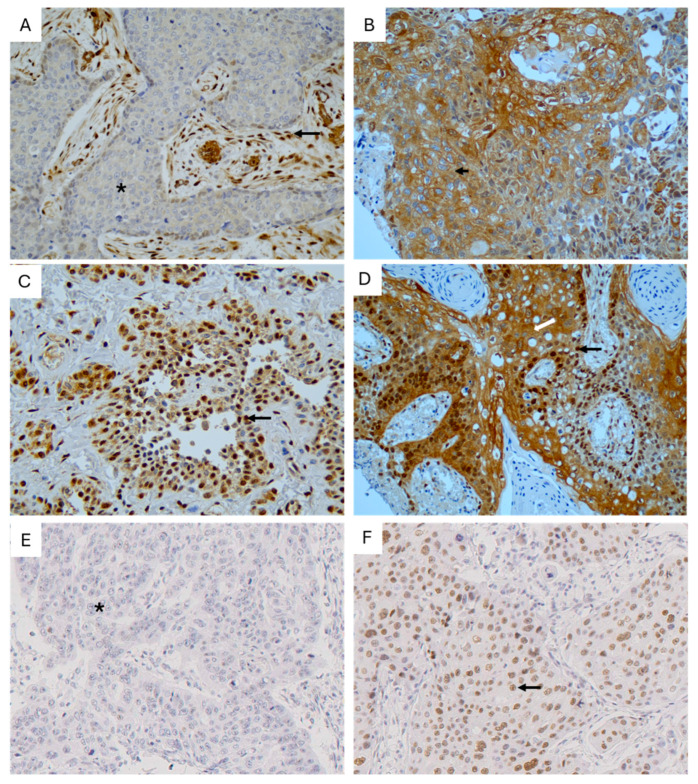
(**A**–**F**). Representative examples of immunohistochemical expression of YAP1 and TAZ proteins in OPSCC tissue specimens. (**A**): YAP1 immunostaining negative in tumor cells (asterisk) and positive in the surrounding stromal fibroblasts (arrow); (**B**): Cytoplasmic (arrow) YAP1-positive tumor; (**C**): Nuclear (arrow) YAP1-positive tumor; (**D**): Nuclear (black arrow) and cytoplasmic (white arrow) YAP1-positive tumor; (**E**): Nuclear TAZ-negative tumor (asterisk); (**F**): Nuclear (arrow) TAZ-positive tumor. Original magnification ×200.

YAP1 staining was also detected within the surrounding tumor stroma, particularly in cancer-associated fibroblasts (CAFs), which showed cytoplasmic and nuclear staining, with moderate/strong expression in 115 tumors (53.5%), weak expression in 88 (40.9%), and absence of staining in 11 cases (5.1%). Representative examples of YAP1 expression are shown in Figure 1A–D.

A significant positive correlation was observed between cytoplasmic and nuclear YAP1 expression (Spearman’s ρ = 0.572, *p* < 0.001). In contrast, stromal YAP1 expression did not correlate with either cytoplasmic or nuclear YAP1 immunostaining in tumor cells.

Nuclear TAZ expression was less frequent, with positive staining (defined as >10% of cells) observed in 31 specimens (14.5%) (Figure 1E,F). One case was not evaluable, resulting in a total of 214 assessable samples for this parameter.

No significant correlation was observed between TAZ and YAP1 expression across any of the evaluated compartments. Specifically, no association was identified at the nuclear level (Spearman’s ρ = 0.04, *p* = 0.56), cytoplasmic level (Spearman’s ρ = 0.10, *p* = 0.12), or within stromal fibroblasts (Spearman’s ρ = 0.11, *p* = 0.09).

### 3.3. Correlation of YAP1 and TAZ Expression with Clinicopathological Parameters and Outcomes

The associations between YAP1 and TAZ expression and clinicopathological parameters are summarized in Table 1. No significant associations were observed between YAP1 or TAZ expression and patients’ sex, tobacco or alcohol exposure, tumor extent (pT), nodal status (pN), or overall clinical stage. Likewise, histological grade was not significantly related to the expression of these markers, although a non-significant trend toward higher nuclear TAZ expression was noted in poorly differentiated tumors. Overall, YAP1 and TAZ expression appeared independent of baseline clinicopathological characteristics.

Regarding disease recurrence, a non-significant trend toward higher nuclear YAP1 expression was observed in patients who developed recurrence compared to those who did not (58% vs. 45%, *p* = 0.07) (Table 2). In contrast, neither cytoplasmic YAP1 nor nuclear TAZ expression showed significant associations with recurrence. When local, regional, and distant recurrences were analyzed separately, a non-significant trend toward a higher proportion of nuclear YAP1–positive cases was observed across all recurrence types (Table 2). A similar pattern was noted for moderate-to-strong cytoplasmic YAP1 expression, although the differences were less pronounced. Conversely, an inverse relationship was observed for TAZ expression, with a lower proportion of TAZ-positive cases among patients with local and regional recurrence (Table 2).

Notably, significant associations emerged when analyzing patient outcomes at last follow-up (Table 1). Nuclear YAP1 expression was significantly higher in patients who died from tumor-related causes (61%) compared to those alive without disease (42%) or those who died from unrelated causes (40%) (*p* = 0.028). A similar pattern was observed for cytoplasmic YAP1 expression, which was more frequent in patients who died from the tumor (50%) compared to those alive without tumor (33%) (*p* = 0.003). In contrast, nuclear TAZ expression was not significantly associated with follow-up status (*p* = 0.21).

Taken together, these findings indicate that YAP1 expression, particularly at both nuclear and cytoplasmic levels, is associated with poorer clinical outcomes, while no consistent associations were observed for TAZ expression with most baseline clinicopathological characteristics or clinical outcomes.

### 3.4. Survival Analysis

**Figure 2:**
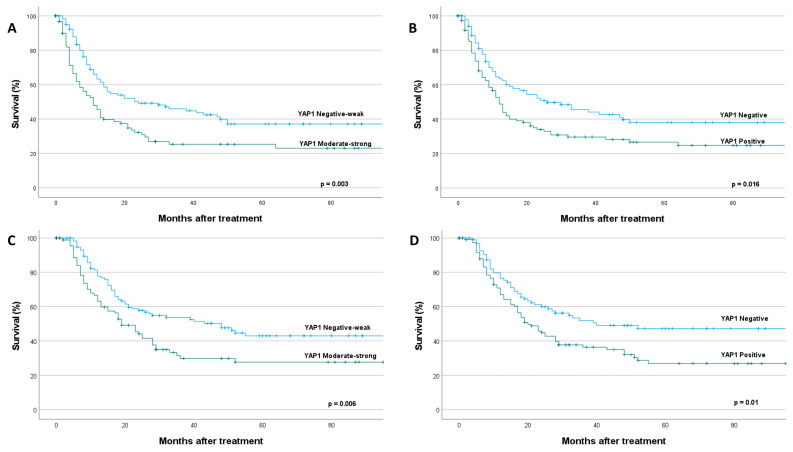
Disease-free survival curves (**A**,**B**) and disease-specific survival curves (**C**,**D**) in relation to cytoplasmic YAP1 expression (**A**,**C**) and nuclear YAP1 expression (**B**,**D**).

Moderate-to-strong cytoplasmic YAP1 expression was significantly associated with reduced disease-free survival (DFS). Median survival was 24 months in the negative/weak expression group compared with 11 months in the moderate/intense expression group (hazard ratio [HR] = 1.67, 95% confidence interval [CI] = 1.18–2.36; *p* = 0.004, log-rank test; Figure 2A). Nuclear YAP1 expression was likewise associated with poorer DFS, with a median survival of 22 months in patients with negative expression versus 12 months in those with positive YAP1 expression (HR = 1.52, 95% CI = 1.07–2.14; *p* = 0.019, log-rank test; Figure 2B). By contrast, no statistically significant differences in DFS were observed according to stromal YAP1 expression (HR = 1.27, 95% CI = 0.89–1.79; *p* = 0.18, log-rank test) or nuclear TAZ expression (HR = 0.9, 95% CI = 0.54–1.53; *p* = 0.72, log-rank test).

Similarly, moderate-to-strong cytoplasmic YAP1 expression was significantly associated with reduced disease-specific survival (DSS). Median survival was 48 months in the negative/weak expression group compared with 19 months in the moderate/intense expression group (HR = 1.63, 95% CI = 1.13–2.35; *p* = 0.006, log-rank test; Figure 2C). Nuclear YAP1 expression was likewise associated with poorer DSS, with a median survival of 40 months in patients with negative expression *versus* 20 months in those with positive YAP1 expression (HR = 1.61, 95% CI = 1.11–2.33; *p* = 0.01, log-rank test; Figure 2D). Again, no statistically significant differences in DSS were observed according to stromal YAP1 expression (HR = 1.24, 95% CI = 0.86–1.79; *p* = 0.24, log-rank test) or nuclear TAZ expression (HR = 0.91, 95% CI = 0.52–1.59; *p* = 0.74, log-rank test).

### 3.5. Multivariable Survival Analysis

Multivariable Cox proportional hazards models were constructed for DFS and DSS, including the following variables: sex, T classification (dichotomized as T1–2 vs. T3–4), N classification (pN0 vs. pN+), histological grade (well/moderately differentiated vs. poorly differentiated), surgical margins status (free vs. involved), administration of postoperative RT (no vs. yes), cytoplasmic YAP1 expression (negative/weak vs. moderate/strong), nuclear YAP1 expression, and TAZ expression. In these models, two variables were associated with a poorer DFS: nodal involvement (HR = 1.64, 95% CI = 1.07–2.54; *p* = 0.023) and positive nuclear YAP1 expression (HR = 1.65, 95% CI = 1.17–2.34; *p* = 0.004); and three variables emerged as independent predictors of poorer DSS: advanced T classification (HR = 1.57, 95% CI = 1.02–2.39; *p* = 0.038), nodal involvement (HR = 2.34, 95% CI = 1.43–3.83; *p* < 0.001), and positive nuclear YAP1 expression (HR = 1.78, 95% CI = 1.23–2.6; *p* = 0.002).

## 4. Discussion

The present study provides a comprehensive evaluation of the Hippo pathway coactivators YAP1 and TAZ in HPV-negative OPSCC, emphasizing the predominant dysregulation and clinical relevance of YAP1 compared with TAZ. Our results demonstrate that YAP1 expression, particularly its nuclear localization (i.e., its activated form), is significantly associated with poorer disease-specific survival and emerges as an independent prognostic factor. In marked contrast, TAZ expression was rather infrequent in this tumor subtype and showed no significant association with clinicopathological parameters or survival outcomes.

### 4.1. YAP1 Activation in HPV-Negative OPSCC

The high prevalence of YAP1 expression observed in our cohort is consistent with the widespread dysregulation of the Hippo pathway in epithelial cancers. Persistent activation of YAP/TAZ signaling is increasingly recognized as a hallmark of HNSCCs, often driven by alterations in upstream regulators rather than direct mutations in YAP1 itself [13,17]. Once translocated to the nucleus, YAP1 functions as a transcriptional coactivator with TEAD factors, promoting the expression of genes involved in proliferation, survival, and stemness [17,18].

An interesting finding from our study is that both cytoplasmic and nuclear YAP1 expression were associated with reduced survival in univariable analysis, although only nuclear YAP1 retained significance in multivariable analysis. This observation deserves careful interpretation, as cytoplasmic YAP1 is generally considered the inactive, sequestered form of the protein. Several biological mechanisms may explain the univariable association between cytoplasmic YAP1 and poor prognosis: First, high cytoplasmic YAP1 expression may represent a large pool of YAP1 protein that can be rapidly mobilized to the nucleus in response to appropriate stimuli, creating potential for dynamic pathway activation. Second, the correlation between cytoplasmic and nuclear YAP1 expression suggests that tumors with high total YAP1 protein levels tend to show both cytoplasmic accumulation and nuclear translocation, with the latter being the functionally dominant event.

The loss of significance for cytoplasmic YAP1 in multivariable analysis when nuclear YAP1 is included suggests that nuclear localization is the functionally critical event for YAP1-driven poor prognosis. This is consistent with the established model of YAP1 regulation, where nuclear translocation enables interaction with TEAD transcription factors and activation of pro-oncogenic gene expression programs.

Our findings linking nuclear YAP1 expression to poor prognosis are in agreement with previous studies in other HNSCC subsites. Rodrigo et al. [10] demonstrated that YAP1 activation correlates with adverse survival outcomes in oral squamous cell carcinomas (OSCC) and is associated with activation of the PI3K pathway [11]. Similarly, YAP has also been implicated in enhancing invasion and metastatic potential in oral cancer, reinforcing its role as a key oncogenic driver [19]. Also, Ge et al. [20] reported that YAP1 overexpression was associated with nodal metástasis in mixed cohorts of OSCC patients. Similarly, Hiemer et al. [21] demonstrated that YAP1 nuclear localization correlated with metastasis and reduced survival in oral squamous cell carcinoma. However, our study is among the first to specifically examine YAP1 in HPV-negative OPSCC, a distinct biological entity.

In addition, recent studies have highlighted the role of YAP1 in regulating chromatin accessibility and transcriptional activity. YAP1 has been shown to cooperate with epigenetic regulators such as BRD4 to maintain an active chromatin state, thereby sustaining tumorigenic transcriptional programs [22]. These findings provide mechanistic support for the association between YAP1 activation and aggressive tumor behavior observed in our cohort.

Moreover, emerging evidence suggests that YAP activity is influenced by biomechanical cues within the tumor microenvironment. Alterations in cellular mechanophenotype and extracellular matrix stiffness can promote YAP nuclear localization and contribute to malignant progression [23]. This reinforces the concept of YAP1 as a central integrator of mechanical and biochemical signals in cancer.

On the other hand, YAP1 is a key mechanosensory transcriptional co-activator that drives the conversion of stromal fibroblasts into cancer-associated fibroblasts (CAFs). In response to extracellular matrix (ECM) stiffening and RhoA–ROCK-mediated cytoskeletal tension, YAP1 accumulates in the nucleus, where it partners with TEAD factors to sustain the activated CAF phenotype through a positive feedback programme [24]. Activated YAP1 promotes expression of cytoskeletal regulators and ECM-remodelling genes, increasing stromal stiffness, enhancing tumour invasion, and limiting drug and immune-cell penetration [24]. It also strengthens tumour–stroma interactions by inducing N-cadherin expression in fibroblasts, thereby activating PI3K–AKT signalling in neighbouring cancer cells [25].

In addition, YAP1-positive CAFs secrete pro-tumorigenic cytokines such as IL-6, IL-8, IL-11, IL-15, and CCL2, supporting angiogenesis, tumour growth, and inflammatory remodelling of the TME [26]. YAP1 also contributes to immune evasion, as YAP1-dependent CAF subsets suppress CD8+ T-cell infiltration and function, reducing responsiveness to immune checkpoint inhibitors [26]. Clinically, elevated stromal nuclear YAP1 is associated with advanced disease and poorer outcomes across several cancer types, highlighting YAP1 as a central regulator of CAF biology and a promising therapeutic target in desmoplastic tumours [27].

It is important to note, however, that in the present cohort, stromal YAP1 expression in CAFs did not reach statistical significance in univariable survival analysis for either DFS (*p* = 0.18) or DSS (*p* = 0.24), and was therefore not entered as a candidate variable in the multivariable prognostic model. While these findings do not negate the biological relevance of stromal YAP1 in the tumor microenvironment, they indicate that its independent clinical prognostic weight in HPV-negative OPSCC requires further investigation in larger, prospectively designed cohorts.

### 4.2. Biological Role of TAZ

In contrast to YAP1, TAZ expression was relatively infrequent and did not demonstrate clinical significance in our cohort. Although YAP and TAZ share structural and functional similarities, accumulating evidence suggests that they may have distinct biological roles depending on tumor context. Interestingly, our results differ from some reports in other cancer types where TAZ shows prominent expression and prognostic value. For example, in gastric cancer [28] and breast cancer [29], TAZ has been reported as a strong prognostic marker. This suggests potential tissue-specific differences in the relative importance of YAP1 versus TAZ, or possible functional redundancy whereby YAP1 is the dominant paralog in OPSCC.

TAZ has been implicated in promoting epithelial–mesenchymal transition (EMT), cancer stem cell maintenance, and metastatic dissemination in HNSCC [30]. Furthermore, TAZ-driven transcriptional activation of SOX2 has been associated with enhanced stemness properties and tumor progression [31]. Stabilization of TAZ protein through deubiquitinating enzymes such as USP7 has also been shown to promote tumor growth and progression [32].

Despite these reported oncogenic functions, our results indicate that TAZ expression is rare and, by itself, does not provide prognostic information in HPV-negative OPSCC. This discrepancy may reflect tumor heterogeneity or suggest that TAZ activity depends on additional molecular events not captured by immunohistochemical analysis. Notably, co-expression patterns, such as TAZ/SOX2 co-localization, have been associated with metastatic behavior in oral cancers [33], indicating that combinatorial biomarkers may be more informative than single-marker evaluation.

### 4.3. Clinical and Therapeutic Implications

While our study is observational and focused on prognostic implications, the strong association between nuclear YAP1 expression and poor survival raises the possibility that YAP1 could represent a potential therapeutic target in HPV-negative OPSCC.

Pharmacological inhibition of YAP/TAZ–TEAD transcriptional activity has shown promising results in preclinical HNSCC models, reducing tumor growth and enhancing therapeutic response [34]. First-in-class YAP/TAZ-TEAD inhibitors entered clinical trials in 2021 [35]. Given the frequent interaction of YAP signaling with other oncogenic pathways, combination approaches targeting both Hippo and MAPK, PI3K, or EGFR pathways may be particularly effective.

Recent advances in the understanding of atypical Hippo signaling networks further support the development of precision medicine strategies in head and neck cancer [17]. In this context, biomarkers such as nuclear YAP1 could help stratify patients most likely to benefit from targeted therapies.

However, functional validation would be required before drawing conclusions about therapeutic vulnerability. Future preclinical studies are warranted to (1) validate YAP1 as a functional driver in HPV-negative OPSCC using in vitro and in vivo models; (2) assess the efficacy of YAP1-targeted therapies in preclinical OPSCC models; and (3) identify potential combination strategies. If validated, YAP1 inhibition could potentially represent a novel therapeutic approach for high-risk HPV-negative OPSCC patients.

Beyond its prognostic value, nuclear YAP1 expression may potentially serve as a predictive biomarker for response to YAP1-targeted therapies. In other cancer types, tumors with high YAP1 activity have shown enhanced sensitivity to YAP1-TEAD inhibitors and certain chemotherapeutic agents [35]. Therefore, nuclear YAP1 assessment could potentially be used to stratify patients for enrollment in clinical trials of YAP1-targeted therapies or to identify those most likely to benefit from such treatments. However, prospective clinical trials with integrated biomarker analysis would be required to validate the predictive value of YAP1 expression. Furthermore, the potential role of YAP1 as a biomarker for response to immunotherapy or conventional chemoradiotherapy warrants investigation, as YAP1 has been implicated in modulating immune responses and treatment resistance in various cancer contexts.

### 4.4. Limitations and Future Directions

This study has several limitations that should be acknowledged. First, its retrospective design may introduce inherent biases. Second, the study population is relatively homogeneous, which may limit the generalizability of the findings. Third, immunohistochemical analysis does not fully reflect the dynamic regulation or functional activation state of YAP/TAZ signaling. It is important to acknowledge that our study assessed YAP1 and TAZ expression and localization by immunohistochemistry, which provides evidence of protein accumulation and nuclear translocation but does not comprehensively evaluate Hippo pathway activity. Nuclear YAP1 localization is widely accepted as a functional readout of pathway activation, as nuclear translocation is required for YAP1 to interact with TEAD transcription factors and drive target gene expression. However, we did not assess upstream Hippo pathway components (*MST1/2*, *LATS1/2*), YAP1 phosphorylation status, pathway mutations, or expression of canonical YAP1 target genes (e.g., *CTGF*, *CYR61*, *ANKRD1*). Therefore, our conclusions regarding Hippo pathway dysregulation are inferred from YAP1 localization patterns rather than direct pathway characterization. Future studies incorporating genomic analysis, assessment of YAP1 phosphorylation, and expression profiling of YAP1 target genes would provide more comprehensive evidence of Hippo pathway dysregulation in HPV-negative OPSCC. Fourth, an important limitation of our study is the lack of external validation in an independent patient cohort. While our cohort of 215 patients is relatively large for HPV-negative OPSCC, validation of the prognostic significance of nuclear YAP1 expression in independent datasets is essential before clinical implementation. We attempted to validate our findings using publicly available head and neck cancer datasets (e.g., TCGA-HNSC); however, these datasets have important limitations: (1) most do not include detailed p16 immunohistochemistry data or separate oropharyngeal from other head and neck subsites; (2) genomic/transcriptomic data may not accurately reflect protein expression and subcellular localization; and (3) treatment paradigms and patient populations may differ substantially. Therefore, prospective validation studies specifically in p16-negative OPSCC cohorts are needed. Multicenter collaborative efforts would facilitate such validation studies.

Based on our findings, we propose the following future directions and recommendations: 1. Clinical validation: External validation of nuclear YAP1 as a prognostic biomarker in independent multi-institutional cohorts of HPV-negative OPSCC patients is essential before clinical implementation. Standardized immunohistochemical protocols and scoring criteria should be established. 2. Mechanistic studies: Functional validation studies using in vitro and in vivo models are needed to confirm YAP1 as a driver of aggressive behavior in HPV-negative OPSCC and to elucidate the molecular mechanisms underlying its prognostic impact. 3. Therapeutic development: Given the strong prognostic significance of nuclear YAP1, preclinical evaluation of YAP1-targeted therapies (e.g., YAP1-TEAD inhibitors) in HPV-negative OPSCC models is warranted. 4. Biomarker integration: Nuclear YAP1 expression could potentially be integrated into prognostic models or risk stratification algorithms to identify high-risk HPV-negative OPSCC patients who may benefit from treatment intensification or enrollment in clinical trials of novel therapies. 5. Molecular profiling: Comprehensive genomic and transcriptomic characterization of YAP1-positive versus YAP1-negative HPV-negative OPSCC would provide insights into pathway activation mechanisms and identify potential combination therapeutic strategies.

These future studies will be critical for translating our findings into clinical applications that improve outcomes for patients with HPV-negative OPSCC.

## 5. Conclusions

Our findings demonstrate that YAP1 plays a central role in the progression of HPV-negative OPSCC and serves as an independent prognostic marker associated with poor disease-specific survival. By contrast, TAZ appears to show rather limited expression and clinical relevance in this context. These results highlight the importance of aberrant YAP1 expression and nuclear localization as a biologically and clinically relevant feature in OPSCC and suggest that YAP1 may represent a potential therapeutic target. External validation in independent cohorts is needed before clinical implementation of YAP1 as a prognostic biomarker.

## Figures and Tables

**Table 1:** Associations of nuclear and cytoplasmic YAP1 and TAZ expression with clinicopathological variables.

Variable	N	Positive Nuclear YAP1 Staining (%)	*p*-Value	Moderate-Strong Cytoplasmic YAP1 Staining (%)	*p*-Value	Positive Nuclear TAZ Staining (%)	*p*-Value
Sex - Men - Women	209 6	113 (54) 1 (17)	0.1	95 (45) 2 (33)	0.69	31 (15) 0	0.31
Tobacco - Yes - No	208 3	111(53) 2 (66)	0.6	96 (46) 1 (33)	0.66	30 (14.5) 1 (33)	0.36
Alcohol - Yes - No	208 3	110 (53) 3 (100)	0.25	94 (45) 3 (100)	0.09	31 (15) 0	0.47
pT classification - T1–2 - T3–4	61 154	31 (51) 83 (54)	0.76	25 (41) 72 (47)	0.45	11 (18) 20 (13)	0.35
pN classification - N0 - N1–N3	54 161	33 (61) 81 (50)	0.2	20 (37) 77 (48)	0.17	10 (18.5) 21 (13)	0.33
Stage - I–II - III - IV	21 36 158	25 (66) 38 (64) 187 (75)	0.204	7 (33) 17 (47) 73 (46)	0.51	4 (19) 6 (17) 21 (13)	0.43
Histologic grade - Low - Moderate - High	96 82 37	11 (52) 23 (64) 80 (50)	0.35	43 (45) 39 (46) 15 (41)	0.77	11 (11.5) 12 (15) 8 (21.5)	0.14
Follow-up status			0.028		0.003		0.21
- Alive without tumor	48	20 (42)		16 (33)		6 (13)	
- Dead from tumor	118	72 (61)		59 (50)		14 (12)	
- Dead from other causes	42	17 (40)		15 (36)		10 (24)	
- Lost	7	5 (71)		7 (100)		1 (14)	
Total	215	114 (53)		97 (45)		31 (14.5)	

**Table 2:** Associations of YAP1 and TAZ expression with tumor recurrence.

Recurrence	Positive Nuclear YAP1 Staining (%)	*p*	Moderate-Strong Cytoplasmic YAP1 Staining (%)	*p*	Positive TAZ Staining (%)	*p*
Local - No - Yes	67 (51%) 47 (57%)	0.4	59 (45%) 38 (46%)	0.87	24 (18%) 7 (8.5%)	0.05
Regional - No - Yes	83 (52%) 31 (57%)	0.46	69 (43%) 28 (52%)	0.25	29 (18%) 2 (4%)	0.01
Distant metastasis - No - Yes	78 (50%) 36 (61%)	0.15	67 (43%) 30 (51%)	0.3	21 (13.5%) 10 (17%)	0.53
Total - No - Yes	38 (45%) 76 (58%)	0.07	33 (39%) 64 (49%)	0.21	15 (18%) 16 (12%)	0.26

## Data Availability

The data presented in this study are available on request from the corresponding author. The data are not publicly available due to privacy and ethical restrictions.

## Abbreviations

The following abbreviations are used in this manuscript:

OPSCC	Oropharyngeal squamous cell carcinoma
HNSCC	Head and neck squamous cell carcinoma
HPV	Human papillomavirus
MST1	Mammalian STE20-like kinases
YAP1	Yes-associated protein
IR	Insulin receptor
DSS	Disease-specific survival
DFS	Disease-free survival
HR	Hazard Ratio
OSCC	Oral squamous cell carcinoma
LSCC	Laryngeal squamous cell carcinoma
